# A simple nonparametric multipoint procedure to test for linkage through mothers or fathers as well as imprinting effects in the presence of linkage

**DOI:** 10.1186/1471-2156-6-S1-S159

**Published:** 2005-12-30

**Authors:** Mathieu Lemire

**Affiliations:** 1 McGill University and Genome Quebec Innovation Centre, and Research Institute of the McGill University Health Centre, Montreal, Quebee, Canada H3A 1A4

## Abstract

A simple multipoint procedure to test for parent-of-origin effects in samples of affected siblings is discussed. The procedure consists of artificially changing all full sibs to half-sibs, with distinct mothers or fathers depending on the parental origin to be evaluated, then analyzing these families with commonly used statistics and software. The procedure leads to tests for linkage through mothers or fathers and also leads to a test for imprinting effects in the presence of linkage. Moreover, simulations illustrate that in regions unlinked to susceptibility genes this multipoint procedure does not have an inflated type I error if a sex-averaged genetic map is used, even when large differences exist between male-specific and female-specific maps. In regions linked with susceptibility genes, the test of imprinting is biased under the null hypothesis if differences exist between sex-specific maps, irrespective of the map used in the analysis. The procedure is applied to the Collaborative Study on the Genetics of Alcoholism dataset from the Genetic Analysis Workshop 14. Results indicate that brothers categorized as affected according to the DMS-III-R and Feighner classification show evidence of linkage through fathers to the 6q25 region (*p *= 0.00038) as well as modest evidence of imprinting (*p *= 0.018). This region harbors *OPRM1*, a candidate gene for substance dependence.

## Background

A simple procedure is proposed to test for linkage through mothers or fathers or to test for imprinting effects in the presence of linkage through the use of nonparametric methods that are based on the estimation of inheritance vectors. This procedure was recently used in an *ad hoc *fashion, without investigating its properties, to evaluate the excess sharing observed from fathers and mothers in a region of chromosome 6 that was suspected to be under the influence of transmission ratio distortion through mothers, in a sample of families selected through asthmatic probands [1]. The procedure I propose is similar in spirit to what has been described in Paterson et al. [2] and Karason et al. [3]. Recently, testing the hypothesis of no linkage in samples of affected sib pairs have been shown to gain power by allowing for differences in penetrances depending on the parental origin of the alleles, when such differences indeed exist [4].

In this paper I investigate some properties of the proposed procedure and apply it to the Collaborative Study on the Genetics of Alcoholism (COGA) dataset.

## Methods

The nonparametric *S*_*pairs *_statistic of Whittemore and Halpern [5] is used as the core statistic to test for linkage. This statistic is transformed into a statistic, *Z*_*lr*_, that is robust against the incompleteness of the marker data, following the method of Kong and Cox [6]. It is asymptotically normally distributed with a mean of 0 and a variance of 1 under the hypothesis of no linkage. Let  be the value of the statistic when applied to nuclear families where the sibs have been artificially recoded as half-sibs with distinct mothers. Then the  statistic evaluates the excess sharing of alleles from fathers, and is a test of linkage through fathers (more precisely it is a test of Mendelian transmission from fathers to affected sibs). Similarly, let  be the value of the statistic when the distinct parents are fathers, which evaluates the sharing of alleles from mothers and is a test of linkage through mothers. I wrote a program, NUCULAR, that splits extended pedigrees into nuclear families with the option of recoding all sibs as half-sibs, with distinct mothers or fathers, who nevertheless maintain their original genetic material.

A test of imprinting effects in the presence of linkage can be constructed by evaluating . This statistic has an approximately normal distribution. The mean of the distribution is 0 and the variance is 1 if the region under study is unlinked to any susceptibility gene (due to independent segregation from both parents), but the variance may be unequal to 1 if there is linkage, because the variance of  and  may be different than 1 and the statistics are likely to be correlated. Consequently, if the region is not linked to any susceptibility genes, the test of imprinting effects has the correct size (but has no power); if the region is linked to a susceptibility gene, the test has power to detect imprinting effects but the true type I error may be different from the nominal one. A solution, if the analysis is carried out using a sex-averaged map, could be a permutation procedure consisting of randomly permuting the genotypes of the parents. This procedure preserves linkage while creating replicates under the null hypothesis of no imprinting effects. It will not correct for the bias introduced if there are large differences in gender-specific genetic maps: the expected values of  and  may then be different under linkage, due to the reduced power of sparser maps to detect linkage [2].

ALLEGRO v1.2 [3,7] was used to carry out the analyses. To investigate the properties of the tests, multipoint gene-dropping simulations were carried out using SIMM [1]. In particular, SIMM allows recombination to occur under sex-specific genetic maps.

I applied the procedure to the COGA microsatellite dataset, using the DMS-III-R and Feighner classification to define the set of affected individuals. Only one nuclear family was extracted per pedigree, the one with the most affected individuals; this is to allow for a valid permutation procedure as described above. This study was approved by the McGill Institutional Review Board.

## Results and Discussion

The type I error of the proposed methods was evaluated in samples of 250 nuclear families with 2 affected sibs by simulating 4 markers located 5 cM (sex-equal) from one another, each having 4 equally frequent alleles (heterozygosity of 0.75), under the rules of Mendelian inheritance. When creating replicates under linkage, an affection status was modelled based on penetrances of 0.18, 0.48, and 0.78 for carriers of 0, 1, or 2 copies of a susceptibility allele found in 20% frequency at a locus located halfway between the second and third marker. In all cases the tests were evaluated halfway between the second and third marker. The same procedure was repeated for sex-specific maps, where the markers were separated by 9 cM in females and 1 cM in males. Results from 10,000 replicates are shown in Table 1 when simulations were performed under sex-equal maps and Table 2 for replicates created under sex-specific maps. All tests show true type I errors close to the nominal ones (none of the estimates are significantly different than their nominal values at the 5% level), even if there were large differences in sex-specific maps. Nevertheless, the  statistic shows a bias when there is linkage and when differences exist between the maps, in that its expectation differs significantly from 0. Note that the bias is not eliminated if the analysis is carried out under the true sex-specific maps. Even if in our case the estimated true type I errors are not significantly different from the nominal ones, the inflation of the type I error is bound to become more important as the sample size increases.

**Figure 1:**
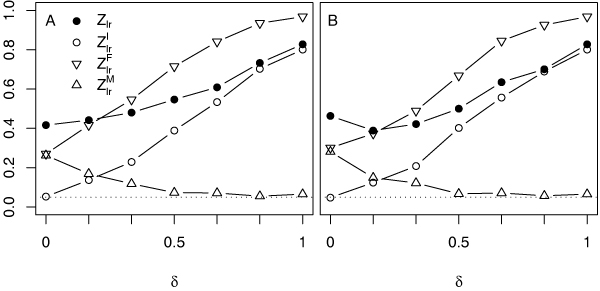
**Power for the models of Table 3**. (A) additive, (B) recessive. Power has been calculated for the test of linkage with full sibs *Z*_*lr *_(solid circles),  (circles),  (triangles point-down),  (triangles point-up). The horizontal dotted line indicates the level of the test (5%). The parameter δ has been scaled to the [0,1] interval, with a value of 1 indicating complete parent-of-origin effects. Power is estimated from 10000 replicates of 250 nuclear families.

For illustration, the power of the tests was computed under two genetic models using the same simulation design as above. The disease locus has two alleles, labelled 1 and 2, with allele 2 conferring susceptibility, found in 20% frequency in the population. Penetrances for the ordered genotype *ij*, where allele *i *is received from the mother, are given in Table 3. The differences in the penetrances of heterozygous individuals are expressed through an imprinting parameter δ to account for parent-of-origin effects. The value is such that there are no parent-of-origin effects when δ = 0 and complete parent-of-origin effects when δ takes its maximum value. Power is evaluated at the position of the disease locus. Results shown in Figure 1 are estimated from 10,000 replicates. The power to detect imprinting in the presence of linkage is very close to the power to detect linkage with full sibs when there are large imprinting effects. Nevertheless, the power to detect imprinting in the presence of linkage is bounded from above by the power to detect linkage. If imprinting is suspected (and knowing the parental source of the increase in risk), then the power to detect linkage is higher using  or  rather than *Z*_*lr *_on the full sibs, unless the imprinting effect is small. Power was also evaluated by simulating the sex-specific maps described above (analyzed assuming a sex-averaged map), and the results are essentially the same. Statistics similar to  and  can be obtained with ALLEGRO v1.2 [3,7], which uses the exact family structure. We repeated the power calculations using these alternative statistics, and the power curves cannot be distinguished from those of Figure 1, indicating that the loss of information that can be expected by ignoring the fact that the sibs truly have the same parents does not reduce power. Although it was not explored here, this statement may not hold if some parents are not genotyped.

Table 4 gives the results of genome-wide tests of linkage through mothers and fathers, as well as tests of imprinting, using the COGA microsatellite data. The genetic contributions to the etiology of alcoholism differ according to sex (e.g., Prescott et al. [8]); thus these tests were performed in affected sibs (105 families, 410 sib pairs), affected sisters (only 35 families, 78 sister pairs), and affected brothers (81 families, 167 brother pairs). For better interpretation of these results, a locus counting approach [9] should be performed to evaluate the effects of multiple testing. Notably, excess sharing of alleles is observed in affected brothers through fathers around marker GATA165G02 in the 6q25.2 region ( = 3.36; *p *= 0.00038), as well as evidence of imprinting ( = 2.36, *p *= 0.018). The significance of the latter result was confirmed by use of the permutation procedure described in Methods; out of 5,000 random replicates that preserve linkage, 1.6% gave an absolute value of  greater than or equal to 2.36 (95% confidence interval for the proportion: 0.0127–0.0199). Approximately 5 cM away from GATA165G02 lies *OPRM1 *(opioid receptor μ-1), a candidate gene for substance dependence [10,11].

Interpretation of tests of imprinting is best if linkage with a susceptibility gene is suspected *a priori*, because without linkage the test has no power. Given prior evidence, the imprinting tests are moreover not bound to small genome-wide significance levels. This is a significant advantage because the power to detect imprinting, seemingly bounded from above by the power to detect linkage, can thus also be quite low for small genome-wide significance levels. The presence of a candidate gene in the 6q25.2 region provides additional strength to the significance of the test of imprinting.

Although *S*_*pairs *_was used as the core statistic, other statistics such as *S*_*all *_[3] or *S*_*ad *_[1] (a statistic that is robust against disease-independent transmission ratio distortion) may be used. The procedure of artificially recoding sibs as half-sibs thus provides flexibility in the choice of the statistic to test for parent-of-origin effects.

## Conclusion

The statistical properties of a simple procedure to test for the presence of parent-of-origin effects were evaluated. Simulations illustrated that the test of imprinting in the presence of linkage is biased under the null when differences exist between male and female genetic maps, irrespective of the map used in the analysis. We found evidence that the 6q25 region may harbor an imprinted susceptibility gene for alcoholism in males.

## Abbreviations

COGA: Collaborative Study on the Genetics of Alcoholism

## Figures and Tables

**Table 1:** True type I error of the tests associated with the nominal values of 1% and 5% for the expected values of the test statistics.

Simulations under sex-equal maps				
Nominal type I error and interval bounds	Unlinked			Linked
	
				

1%	0.0099	0.0144	0.0084	0.0105
5%	0.0511	0.0500	0.0478	0.0495
Lower Cl	**-0.0099**^a^	**-0.0141**	**-0.0221**	**-0.0354**
Upper Cl	**-0.0289**	**0.0251**	**0.0164**	**0.0038**

^a^Bold indicates 95% confidence interval boundary.

Simulations were performed in a region unlinked to any susceptibility gene and a region linked to a susceptibility gene.

**Table 2:** Simulations performed with a sex-averaged map and the sex-specific map for the replicates linked to a susceptibility gene.

Simulations under sex-specific maps					
Nominal type I error and interval bounds				Linked	
				
	Unlinked			Averaged map used	Specific map used
		
					

1%	0.0112	0.0099	0.0104	0.0089	0.0107
5%	0.0524	0.0503	0.0516	0.0512	0.0462
Lower Cl	**-0.0070**^a^	**-0.0025**	**-0.0129**	**0.0509**	**0.0608**
Upper Cl	**0.0325**	**0.0417**	**0.0262**	**0.0900**	**0.0998**

^a^Bold indicates 95% confidence interval boundary.

**Table 3:** Sets of penetrances *f*_*ij *_for the ordered genotype *ij*.

Genetic models for power calculations				
Model	*f*_11_	*f*_21_	*f*_12_	*f*_22_

Additive	0.18	0.48 - δ	0.48 + δ	0.78
Recessive	0.18	0.18	0.18 + δ	0.78

Differences in the penetrances of heterozygous individuals are expressed through a parameter δ that ranges from 0 to 0.3 for the additive model and from 0 to 0.6 for the recessive model. For the penetrance *f*_ij_, allele *i *is received from the mother.

**Table 4:** Tests of linkage through fathers (), mothers (), and a test of imprinting () in affected sibs, affected sisters and affected brothers.

Testing parent-of-origin effects in the COGA dataset								
Pair type (number of sibships)	Chr	cM		*p*		*p*		*p*

Affected sibs (105)	11	127.814	3.17	0.00076	0.78	0.22	1.69	0.092
Affected sisters (35)	21	26.27	2.10	0.18	3.21	0.00067	-0.78	0.44
Affected brothers (81)	6	160.33	3.36	0.00038	0.024	0.49	2.36	0.018

Results are reported if one test has p<0.001.
